# Executive Functioning, Muscle Power and Reactive Balance Are Major Contributors to Gait Adaptability in People With Parkinson’s Disease

**DOI:** 10.3389/fnagi.2019.00154

**Published:** 2019-06-28

**Authors:** Maria Joana D. Caetano, Stephen R. Lord, Natalie E. Allen, Jooeun Song, Serene S. Paul, Colleen G. Canning, Jasmine C. C. Menant

**Affiliations:** ^1^Independent Researcher, São Carlos City Hall, São Carlos, Brazil; ^2^Neuroscience Research Australia (NeuRA), Sydney, NSW, Australia; ^3^School of Public Health & Community Medicine, University of New South Wales, Sydney, NSW, Australia; ^4^Faculty of Health Sciences, The University of Sydney, Sydney, NSW, Australia

**Keywords:** Parkinson’s disease, gait adaptability, obstacle avoidance, cognition, choice stepping reaction time, stroop stepping test

## Abstract

**Background and Aim**: The ability to adapt gait when negotiating unexpected hazards is crucial to maintain stability and avoid falling. This study investigated cognitive, physical and psychological factors associated with gait adaptability required for obstacle and stepping target negotiation in people with Parkinson’s disease (PD).

**Methods**: Fifty-four people with PD were instructed to either: (a) avoid an obstacle at usual step distance; or (b) step onto a target at either a short or long step distance projected on a walkway two heel strikes ahead and then continue walking. Participants also completed clinical [Hoehn & Yahr rating scale; Movement Disorders Society version of the Unified Parkinson’s Disease Rating Scale motor section (MDS-UPDRS-III)], cognitive [simple reaction time, Trail Making and Stroop stepping (difference between incongruent and standard Choice Stepping Reaction Time, CSRT) tests], physical [hip abductor muscle power and reactive balance (pull test from the MDS-UPDRS-III)] and psychological (Fall Efficacy Scale–International) assessments.

**Results**: Discriminant function analysis revealed Stroop stepping test (inhibitory control) performance was the best predictor of stepping errors across the Gait Adaptability Test (GAT) conditions. Poorer executive function [Trail Making Test (TMT)] and reactive balance predicted poorer stepping accuracy in the short target condition; poorer reactive balance predicted increased number of steps taken to approach the obstacle and the long target; and poorer executive function predicted obstacle avoidance. Weaker hip abductor muscle power, poorer reactive balance, slower reaction time, poorer executive function and higher concern about falling were significant predictors of shorter step length while negotiating the obstacle/targets.

**Conclusion**: Superior executive function, effective reactive balance and good muscle power were associated with successful gait adaptability. Executive function and reactive balance appear particularly important for precise foot placements; and cognitive capacity for step length adjustments for avoiding obstacles. These findings suggest that impaired inhibitory control contributes to stepping errors and may increase fall risk in people with PD. These findings help elucidate mechanisms for why people with PD fall and may facilitate fall risk assessments and fall prevention strategies for this group.

## Introduction

The incidence of falls in people with Parkinson’s disease (PD) is higher than in the healthy older population. Prospective studies indicate that between 45%–68% of people with PD fall at least once a year (Wood et al., 2002; Pickering et al., 2007; Latt et al., 2009; Paul et al., 2013; Lamont et al., 2017), with a large proportion (39%) falling recurrently (Allen et al., 2013). Most falls occur when people with PD are walking (Mak and Pang, 2010) and when they are optimally medicated (Gray and Hildebrand, 2000; Bloem et al., 2001; Lamont et al., 2017). It is possible that declines in the ability to adapt gait behavior, particularly under challenging environmental conditions contribute to trips; which are a frequently reported cause of falls in people with PD (Mak and Pang, 2010; Stack and Roberts, 2013; Gazibara et al., 2014).

Several studies have identified spatiotemporal gait alterations in people with PD walking at self-selected comfortable speed whilst optimally medicated. Compared with controls, people with PD walk slower with shorter stride length (Lewis et al., 2000; Sofuwa et al., 2005; Yang et al., 2008; Caetano et al., 2009) and slower cadence (Morris et al., 1994), present an increased double support duration (Caetano et al., 2009), more variable stride time (Hausdorff et al., 1998; Lord et al., 2013) and reduced foot clearance (Alcock et al., 2016). PD-related gait alterations interfere with the performance of daily activities, particularly in challenging conditions requiring modification of the walking pattern to deal with environmental changes or other task demands. Indeed, there is evidence that the ability to make gait adjustments in response to upcoming environmental changes is impaired in PD (Galna et al., 2010, 2013; Vitório et al., 2010, 2016; Stegemoller et al., 2012; Pieruccini-Faria et al., 2013; Geerse et al., 2018).

Several studies have used obstacle avoidance and stepping target tasks to assess gait adaptability in people with PD. This work has shown that compared with control participants, people with PD walk slower and take shorter steps throughout the approach, crossing and recovery steps of obstacle crossing (Galna et al., 2010; Vitório et al., 2010; Stegemoller et al., 2012; Pieruccini-Faria et al., 2013). Further, people with PD have reduced foot clearances (shorter vertical foot-obstacle distance), poorer balance control (increased speed and sway of the center of mass; Galna et al., 2013) during the obstacle crossing and place the lead foot closer to the obstacle after crossing it (Galna et al., 2010; Vitório et al., 2010; Stegemoller et al., 2012). People with PD also exhibit impaired foot placement accuracy in a walking task involving fixed stepping targets (Vitório et al., 2016; Geerse et al., 2018) and display lower obstacle-avoidance success rates and smaller obstacle-foot distances when adapting their walking to suddenly appearing obstacles (Geerse et al., 2018).

We recently designed a gait adaptability task to simulate the motor and cognitive challenges of daily walking activities. This task required people to either step onto a target or avoid an obstacle appearing at short notice while walking on a pathway in the laboratory. A decision-making component was incorporated into the walking task by using two stimulus colors (pink and green) that triggered different responses: a pink stimulus required an avoidance strategy (obstacle) whereas a green stimulus required a stepping strategy (target). We found that people with PD had more difficulty adapting their gait in response to targets (poorer stepping accuracy) and obstacles (increased number of steps) appearing at short notice on a walkway in comparison with healthy control participants (Caetano et al., 2018a). We noted such gait impairments were related to PD symptoms (Caetano et al., 2018a), but did not investigate whether gait adaptability deficits were also related to specific physical and cognition related impairments.

In the current study, we aimed to identify cognitive, physical and psychological factors associated with gait adaptability in people with PD. Seven variables related to falls in PD (Latt et al., 2009; Allen et al., 2010; Kerr et al., 2010; Paul et al., 2013, 2014) were examined: (i) freezing of gait; (ii) concern about falling; (iii) reactive balance (retropulsion test); (iv) lower limb muscle power; (v) simple reaction time; (vi) set-shifting of executive function; and (vii) inhibitory control of executive function. Our primary hypothesis was that cognitive capacities would discriminate between PD participants who do and do not make errors in the Gait Adaptability Test (GAT). Our secondary hypothesis was that a combination of cognitive and physical factors would predict stepping parameters in the experimental gait adaptability trials.

## Materials and Methods

### Participants and Ethics Approval

The sample comprised 54 people with PD who were recruited from metropolitan Sydney, Australia through the research team’s research volunteer databases and through Parkinson’s NSW newsletters and support groups. PD volunteers were recruited for a training study (ACTRN12613000688785) and their data were collected as part of the baseline assessments. Participants were included if they were living in the community, able to walk unaided for ≥30 m and cognitively capable of following all instructions (MOCA scores ≥20; Nasreddine et al., 2005). Participants were required to have been on the same PD medication for at least 2 weeks. Volunteers were excluded if they had any medical conditions which would preclude or interfere with the physical assessment (e.g., physician diagnosed dementia, acute or terminal illness, progressive neurodegenerative diseases (other than PD), major psychiatric illnesses, color-blindness or visual impairments that could not be corrected). The University of Sydney Human Research Ethics Committee approved this study and all participants gave informed consent prior to study participation.

All measurements were conducted while participants were “on” their usual PD medication. Researchers experienced in working with people with PD and trained in the Movement Disorders Society Unified Parkinson’s Disease Rating Scale (MDS-UPDRS) administered section III of the scale (motor examination; Goetz et al., 2012), the Hoehn & Yahr rating scale (H&Y; Hoehn and Yahr, 1967) and The New Freezing of Gait Questionnaire (NFOG-Q, part I: dichotomous item in which individuals were classified as a freezer or a non-freezer if they had experienced freezing of gait episodes during the past month; Nieuwboer et al., 2009). Psychological assessment regarding participants’ concern about falling was determined with the Fall Efficacy Scale–International (Yardley et al., 2005).

### Protocol

Participants performed the GAT as well as brief cognitive and physical capacity assessments.

#### Gait Adaptability Test (GAT)

Participants wore their own comfortable flat shoes and were required to walk at their self-selected speed over an obstacle-free path (baseline condition). They were then instructed about the GAT. As described elsewhere (Caetano et al., 2017), the GAT required participants to complete walking trials in four experimental conditions: (i) avoid stepping on a pink stimulus appearing two steps ahead (obstacle avoidance); (ii) stepping onto a green stimulus appearing slightly short of two steps ahead (short target); (iii) stepping onto a green stimulus appearing slightly further than two steps ahead (long target); and (iv) walking with no stimulus appearing on the pathway (walk-through). Walk-through trials were included to encourage participants to walk naturally. Trials were presented in a randomized order for a total of three trials per condition. At least one practice trial per condition (baseline, walk-through, obstacle avoidance, short target and long target) was performed until the task was understood, before data acquisition. Participants were offered rest breaks throughout the test.

**Figure 1:**
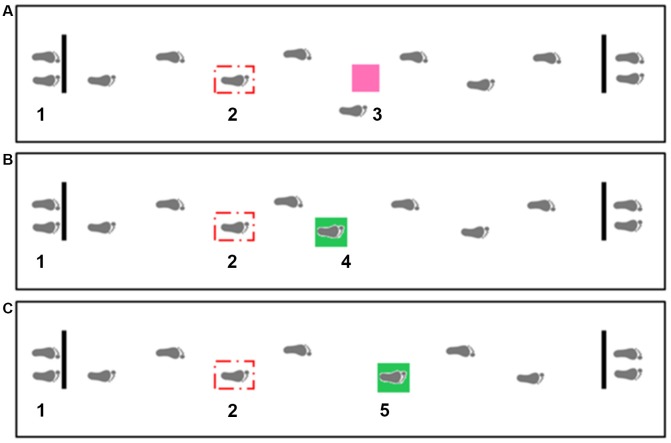
Overhead view of the gait adaptability experimental setup including obstacle avoidance **(A)**, short target **(B)** and long target **(C)** conditions. Distance to the obstacle/target was personalized for each individual. The starting position (1) was adjusted to align the obstacle (3) with the fifth-foot landing location based on the average foot placement from the baseline walking trials. The stepping targets were projected in two locations −24.5 cm anterior (4) and 24.5 cm posterior (5) to the obstacle position (center to center distance), and thus required a short or a long step length respectively. The projection system for the three stimulus consisted of three torches installed in the ceiling and connected to a control box. A force sensitive resistor (Sparkfun SEN-09376) placed underneath the participant’s right shoe and connected to a wireless transmitter attached to the participant’s ankle triggered the light projection on the third heel strike following gait initiation (2).

The equipment and set-up have also been described in detail previously (Caetano et al., 2017). In brief, the targets and obstacle consisted of a colored light stimulus projected onto an area on the walkway (23 × 23 cm), presented on the third heel strike following gait initiation and appearing two steps ahead of the participant (Figure 1). Participants were instructed to step in the middle of the targets (green light) and to avoid stepping on the obstacle (pink light) but not step off the mat, using any avoidance strategy. Participants were asked to start walking with the right foot in all conditions. Distance to the obstacle/target was personalized for each individual. The starting position was adjusted to align the obstacle with the fifth-foot landing location based on the average foot placement from the baseline walking trials. During the experimental trials, obstacle and targets were presented on the third heel strike following gait initiation. In the trials where participants maintained their gait pattern similar to the baseline, the obstacle/targets appeared at two-step distance. However, it was a common behavior that participants adjusted their gait parameters after the appearance of the obstacle/targets. Considering our aims were to identify gait adaptation strategies toward suddenly appearing obstacles and targets, we consciously decided to not adjust the obstacle/target location for each experimental trial, but rather included the number of steps taken while approaching the obstacle/target as a dependent variable.

For the purpose of understanding stepping strategies, the step that hit or avoided the stimulus was named “target/obstacle step” and the preceding step was named “previous step.” Step length values were normalized by height of the participants. The average of the successful trials per condition was used in the analysis. Gait adaptability performance outcome measures were: (i) GAT errors—number of participants who made at least one error (stepping on an obstacle or missing a target); (ii) stepping accuracy for short and long target conditions (distance between the center of the target and the center of the foot); (iii) number of steps taken to approach the target or obstacle (during interval between the appearance of the stimulus and the target or obstacle step); and (iv) length of the two steps preceding the target or obstacle. An electronic walkway (4 m-long ZenoMetrics^®^mat/PKMAS software, v2011–2013, Havertown, PA, USA) recorded the temporal and spatial gait parameters. Position coordinates of the foot with reference to the target or obstacle coordinates extracted from the electronic walkway were used to determine GAT variables using a Matlab routine (MathWorks, Natick, MA, USA).

#### Cognitive Assessments

Cognitive capacity was assessed using a test of simple reaction time (Lord et al., 2003), the Trail Making (Lezak et al., 2004) and the Stroop Stepping (Schoene et al., 2011, 2014) tests. For the assessment of simple reaction time (processing speed), participants were seated at a table and asked to press a button of a modified computer mouse using the index finger of their dominant hand as quickly as possible when a light stimulus appeared (Lord et al., 2003). Five practice trials were undertaken, followed by 10 experimental trials, with the average time of the experimental trials calculated in milliseconds.

The Trail Making Test (TMT) evaluates the cognitive flexibility/set-shifting of executive function [difference in execution time between parts B and A (TMT score)]. Participants were instructed to connect consecutive circled numbers for the TMT—part A and to connect numbers and letters in an alternating sequence for the TMT—part B, as quickly as possible without lifting the pen from the article. If participants made an error they were informed immediately and allowed to correct it. The total time to complete each part was measured in seconds with the test time capped at 5 min.

**Figure 2:**
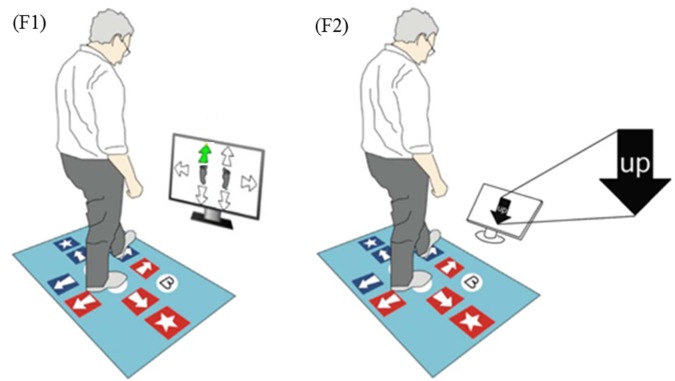
Stepping tests used in this study. **(F1)** Choice stepping reaction time (CSRT) test example screen. One of six arrows on the screen changes its color to green and the participant is asked to step as quickly as possible onto the same location of the pad (front left in this example). **(F2)** Stroop Stepping test example screen. Participants step according to the word and not the arrow orientation.

Inhibitory control of executive function was measured with the Choice Stepping Reaction Time (CSRT) and the Stroop Stepping tests (Schoene et al., 2011, 2014) using a custom-made step mat (Figure 2). Descriptions of the apparatus, procedures and test-retest reliability for these tests are reported elsewhere (Schoene et al., 2011, 2014; Caetano et al., 2018a). In brief, the CSRT test assesses the ability to take a rapid step forward, backward or sidewards with either leg in response to randomly presented stimuli (Schoene et al., 2011). Six practice trials and 18 test trials were administered for this test. The Stroop stepping test combines stepping and response inhibition requiring rapid responses to incongruent stimuli (Schoene et al., 2014). A random sequence of four practice trials and 20 test trials in which the directions of word and orientation never matched was administered. In both tests, the average response time (i.e., stimulus presentation to step-on the target) was measured in milliseconds (ms). In the present study, the CSRT test was used as a proxy for a congruent Stroop Stepping test and the difference in execution time between both tests (Stroop stepping score) was used as a measure of inhibitory control.

#### Physical and Balance Assessments

Physical and balance assessments included tests of hip abductor muscle power and reactive balance. Hip abductor muscle power was measured in Watts for each leg using pneumatic variable resistance equipment (Keiser A420, Keiser Sports Health Equipment, Fresno, CA, USA). Muscle power was measured by having the participant abduct the hip as fast as possible against a low load (35 N—equivalent to 30% of the one repetition maximum on average in people with PD; Paul et al., 2012). Muscle power was recorded as the average of both legs.

Reactive balance was measured using the retropulsion test from the MDS-UPDRS Rating Scale (item 3.12; Goetz et al., 2012). The test examines the response to sudden body displacement produced by a quick, forceful pull on the shoulders while the patient is standing erect with eyes open and feet comfortably apart and parallel to each other. Performance was rated with a 0 (Normal: No problems, recovers with one or two steps), 1 (Slight: 3–5 steps, but subject recovers unaided), 2 (Mild: More than 5 steps, but subject recovers unaided), 3 (Moderate: Stands safely, but with absence of postural response; falls if not caught by examiner) or 4 (Severe: Very unstable, tends to lose balance spontaneously or with just a gentle pull on the shoulders).

### Statistical Analysis

Data normality was confirmed using the Skewness test. Discriminant function analysis was used to determine which cognitive, physical and psychological variables discriminated between the PD participants who made one or more mistakes in the GAT from those who did not; Only those variables that were statistically and independently associated with gait adaptability errors were retained in the final model. Discriminant function analysis was chosen because our outcome was dichotomous and all the putative predictor variables were continuously scaled. Pearson’s correlations were computed to examine associations between the cognitive, physical and psychological measures and the gait adaptability parameters. Stepwise linear regression analyses were then performed to identify independent and significant cognitive, physical and psychological explanatory variables for stepping accuracy in the short and long target conditions; the number of steps in the short target, long target and obstacle avoidance conditions and step length of the previous and target/obstacle steps for all conditions. The cognitive, physical and psychological variables that showed the strongest significant correlations with the gait adaptability measures were entered as standardized z-scores into the stepwise linear regression with a limitation of one predictor variable per 10 cases. All statistical analyses were performed using SPSS (Version 25 for Windows, SPSS Science, Chicago, IL, USA), with significance set at *p* < 0.05.

## Results

### Gait Adaptability Performance

Table 1 shows demographic, clinical, cognitive, physical and psychological measures for the sample, and Table 2 presents participants’ performance data for the gait adaptability variables for each test condition. Fourteen participants with PD (26%) made at least one error in the experimental conditions, totaling 18 incorrect responses of which 13 were commission errors (step on the obstacle) and five were omission errors (avoid the target—all in the short target condition).

**Table 1:** Anthropometric, clinical, cognitive and physical characteristics of participants with Parkinson’s disease (*n* = 54).

Age (years)	66 (7)
Gender (male)	30 (56%)
Body Height (m)	1.7 (0.1)
Body Weight (kg)	76 (16)
Cognitive Status MoCA (score 0–30)	26 (2.6)
Previous falls, number of participants, yes (%)	29 (54%)
Duration of Parkinson’s disease since diagnosis (years)	7.8 (5.1)
Hoehn & Yahr stage	2.0 (0.5)
Disease severity “On” MDS-UPDRS Part III (score 0–132)	30 (11)
Freezing of Gait (NFOG-Q), number of participants, yes (%)	16 (30%)
Concern about falling (FESI, score 16–64)	25.5 (8.6)
TMT score (s)	50.9 (37.5)
Stroop stepping score (ms)	1177 (491)
Simple reaction time (ms)	256 (58)
Hip abductor muscle power (w)	44.3 (17.2)
Reactive balance (score 0–4)	1 (1)

*Data are mean (SD) or number (%). MoCA, Montreal Cognitive Assessment; MDS-UPDRS, Movement Disorders Society version of the Unified Parkinson’s Disease Rating Scale; NFOG-Q, New freezing of gait questionnaire (part I, yes/no); FESI, Fall Efficacy Scale–International; TMT score: Trail Making Test, time difference between part B and part A; Stroop stepping score: time difference between Choice Stepping Reaction Time and Stroop Stepping tests. *Note*: higher scores in concern about falling, TMT, Stroop stepping, simple reaction time and reactive balance tests mean worse performance, while higher scores in MoCA and muscle power mean better performance*.

**Table 2:** Gait adaptability test variables for the Parkinson’s disease participants (*n* = 54).

Errors [# of participants (%)]^a^	14 (26%)
Stepping accuracy (cm)^b^	
Short target	7.7 (4.1)
Long target	7.7 (3.9)
Number of steps^c^	
Short target	2.2 (0.5)
Long target	2.9 (0.6)
Obstacle avoidance	2.9 (0.6)
Step length (m)^d^	
Baseline	0.69 (0.11)
Walk-through	0.64 (0.13)
Short target	
Previous step	0.56 (0.16)
Target step	0.58 (0.14)
Long target	
Previous step	0.57 (0.15)
Target step	0.64 (0.16)
Obstacle avoidance	
Previous step	0.54 (0.17)
Obstacle step	0.71 (0.18)

Data presented are mean (SD) unless stated otherwise. ^a^Number of participants (%) who made at least one mistake in the gait adaptability test. ^b^Distance between the center of the target and the center of the foot; high values mean worse performance. ^c^Number of steps taken to approach the target or obstacle (during interval between the appearance of the stimulus and the target or obstacle step). ^d^Step that hit or avoided the stimulus was named “target/obstacle step” and the preceding step was named “previous step.”

### Predictors of Impaired Gait Adaptability

Discriminant function analysis identified Stroop stepping performance as the only independent and significant predictor of stepping errors across the GAT conditions (Wilk’s lambda: 0.822, *p* < 0.003; canonical correlation: 0.422).

Several cognitive, physical and psychological factors were weakly to moderately correlate with many gait adaptability performance measures (Table 3). The stepwise linear regression models revealed: (i) poorer reactive balance and executive function (TMT performance) were independent and significant predictors of poorer stepping accuracy in the short target condition; and (ii) poorer reactive balance (long target and obstacle avoidance conditions) and executive function (obstacle avoidance condition) were independent and significant predictors of increased number of steps taken to approach the target/obstacle (Table 4).

**Table 3:** Correlations coefficients among gait, clinical, cognitive, physical and psychological variables for each condition: baseline, walk-through, short target, long target and obstacle avoidance.

	Cognitive			Physical		Psychological	Clinical
Variables	TMT score^a^	Stroop stepping score^b^	Simple reaction time	Muscle power	Reactive balance	Concern about falling	Freezing of gait^c^
**GAT error**	0.319*	0.450**	0.392**	−0.328	0.184	0.062	0.079
**Stepping accuracy (cm)**
Short target	0.346*	0.335*	0.057	−0.271*	0.425**	0.286*	0.211
Long target	0.022	0.143	0.072	−0.041	0.102	0.196	0.315*
**Number of steps**
Short target	−0.037	0.169	−0.065	−0.125	0.190	0.066	−0.132
Long target	0.128	0.80	0.267	−0.175	0.320**	0.196	−0.158
Obstacle	0.309*	0.219	0.113	−0.137	0.437**	0.268*	−0.039
**Step length (m)**
Baseline	−0.184	−0.434**	−0.299*	0.689**	−0.457**	−0.466**	−0.266
Walk-through	−0.226	−0.498**	−0.327*	0.660**	−0.477**	−0.461**	−0.176
Short target
Previous step	−0.149	−0.400**	−0.141	0.525**	−0.458**	−0.370**	−0.101
Target step	−0.103	−0.470**	−0.092	0.484**	−0.372**	−0.253	−0.242
Long target
Previous step	−0.270	−0.225	−0.421**	0.438**	−0.466**	−0.352**	−0.076
Target step	−0.128	−0.212	−0.269*	0.390**	−0.428**	−0.277*	−0.006
Obstacle
Previous step	−0.369**	−0.395**	−0.193	0.361**	−0.367**	−0.306*	−0.029
Obstacle step	−0.181	−0.449**	−0.225	0.421**	−0.342*	−0.284*	−0.165

Note: higher scores in TMT, Stroop stepping, simple reaction time and reactive balance tests mean worse performance, while higher scores in muscle power mean better performance. Increased stepping accuracy values indicate poorer performance. ^a^Trail Making Test, time difference between part B and part A. ^b^Time difference between Choice Stepping Reaction Time and Stroop Stepping tests. ^c^New freezing of gait questionnaire (NFOG-Q, Part 1, yes/no). *Significant correlation (*p* < 0.05), **Significant correlation (*p* < 0.01).

**Table 4:** Stepwise linear regression models for predicting stepping accuracy in the short target and long target conditions, number of steps in the short target, long target and obstacle avoidance conditions and step length in the baseline, walk-through, short target, long target and obstacle avoidance conditions.

Variables	Model result	Explained variance	Significant predictors	
**Stepping accuracy**
Short target	*F*_(2,52)_ = 8.864, *p* = 0.001	26%	Reactive balance	*β* = 0.381, *p* = 0.003
			TMT score	*β* = 0.290, *p* = 0.022
Long target	-	-	None identified
**Number of steps**
Short target			None identified
Long target	*F*_(1,53)_ = 5.933, *p* = 0.018	10%	Reactive balance	*β* = 0.320, *p* = 0.018
Obstacle	*F*_(2,52)_ = 8.645, *p* = 0.001	26%	Reactive balance	*β* = 0.249, *p* = 0.046
			TMT score	*β* = 0.406, *p* = 0.009
**Step length**
Baseline	*F*_(2,53)_ = 27.193, *p* < 0.001	52%	Muscle power	*β* = 0.598, *p* < 0.001
			Concern about falling	*β* = −0.223, *p* = 0.042
Walk-through	*F*_(2,53)_ = 23.708, *p* < 0.001	48%	Muscle power	*β* = 0.558, *p* < 0.001
			Reactive balance	*β* = −0.239, *p* < 0.001
Short target
Previous step	*F*_(2,53)_ = 13.306, *p* < 0.001	34%	Muscle power	*β* = 0.403, *p* = 0.002
			Reactive balance	*β* = −0.287, *p* = 0.027
Target step	*F*_(2,53)_ = 11.429, *p* < 0.001	31%	Muscle power	*β* = 0.338, *p* = 0.013
			Stroop stepping score	*β* = −0.311, *p* = 0.022
Long target
Previous step	*F*_(2,53)_ = 11.650, *p* < 0.001	31%	Reactive balance	*β* = −0.383, *p* = 0.002
			Simple reaction time	*β* = −0.321, *p* = 0.010
Target step	*F*_(1,53)_ = 11.640, *p* = 0.001	18%	Reactive balance	*β* = −0.428, *p* = 0.001
Obstacle
Previous step	F_(3,52)_ = 7.562, *p* < 0.001	32%	Stroop stepping score	*β* = −0.276, *p* = 0.029
			TMT score	*β* = −0.332, *p* = 0.008
			Concern about falling	*β* = −0.282, *p* = 0.023
Obstacle step	*F*_(1,53)_ = 13.138, *p* = 0.001	20%	Stroop stepping score	*β* = −0.449, *p* = 0.001

Further, independent and significant physical predictors of shorter step length were weaker hip abductor muscle power (baseline, walk-through and previous and target steps in the short target condition) and poorer reactive balance (walk-through, previous step in the short/long target conditions and target step in the long target condition). Independent and significant cognitive predictors were slower simple reaction time (previous step in the long target condition), poorer Stroop stepping (target step in the short target condition and previous and obstacle steps in the obstacle avoidance condition) and TMT performances (previous step in the obstacle avoidance condition). Finally, greater concern about falling was an independent and significant predictor of shorter step length (baseline and previous step in the obstacle avoidance condition; Table 4).

## Discussion

This study examined associations between cognitive, physical and psychological factors and gait adaptability in people with PD. Our hypotheses were supported by the findings of significant associations between successful gait adaptability and intact cognition as assessed with the trail making, Stroop stepping and simple reaction time tests in addition to better hip abductor muscle power and reactive balance.

### Cognitive Correlates

Inhibitory control of executive function (Stroop stepping score) was identified as the variable that best discriminated between participants who did and did not make mistakes (i.e., failing to hit the stepping targets or avoid the obstacle) in the GAT. Stroop stepping performance was also a predictor of target step length in the short target condition and the most powerful predictor for the obstacle avoidance condition (previous and obstacle step lengths). These results are in line with our previous study in healthy older adults (Caetano et al., 2017) and confirm the importance of inhibitory control of executive function for gait adaptability, particularly in people with PD. Our findings are also consistent with a previous study that found people with PD, compared with controls, make more errors in the incongruent trials of the finger-tapping Stroop task (Vandenbossche et al., 2012), and evidence that attentional control deficits lead to less effective behavioral responses in people with PD (Cools et al., 2010).

The identification of Stroop stepping performance as a predictor for both short target and obstacle avoidance conditions reflects the inhibitory component of the walking task that required participants to select the appropriate response while suppressing a dominant one (Caetano et al., 2017). Inhibitory control is an important discriminator between fallers and non-fallers in healthy older people (Anstey et al., 2009; Mirelman et al., 2012), and gait adaptability mistakes are more prevalent among older people at high risk of falling (Caetano et al., 2018b). Thus, our results suggest that impaired inhibitory control of executive function contributes to poor gait adaptability and consequently increased fall risk among people with PD.

Cognitive flexibility of executive function (TMT score) predicted stepping accuracy in the short target condition. Considering people with PD are more dependent on visual feedback to make target steps (Vitório et al., 2016), the short target condition appears to have been more challenging than the long target condition due to the shorter response time available to identify the target and plan and execute the appropriate gait adjustments. TMT performance was significantly associated with the number of steps taken to approach the obstacle and previous step length in the obstacle condition, suggesting that gait adjustments for obstacle avoidance require earlier and/or additional cognitive processing than stepping onto a target, consistent with findings in healthy older adults (Caetano et al., 2017).

Further, slow reaction time was significantly associated with shorter previous step length in the long target condition. Taken together, the above findings suggest that intact cognition is important for safe and precise foot placement for fall avoidance and may elucidate why reduced cognitive performance has been strongly associated with falls in people with PD (Paul et al., 2014).

### Muscle Power, Reactive Balance and Concern About Falling

We found reduced muscle power was associated with short step length in the baseline, walk-through and short target conditions; findings that build on previous work that has shown people with PD have shorter step lengths during usual (Sofuwa et al., 2005) and adaptive (Vitório et al., 2010) gait as well as reduced lower limb muscle strength and power than healthy controls (Allen et al., 2009). Robichaud et al posited that disease-related changes in the organization of the basal ganglia-thalamo-cortical circuit produce abnormal force generation patterns (shorter agonist burst duration and delayed antagonist activation; Robichaud et al., 2002) and subsequent shorter steps, and this may be exacerbated in a walking condition requiring gait adaptability. Thus, a multiple short step strategy in our gait adaptability protocol may have been adopted to compensate for motor deficits (Jankovic, 2008) or indicate difficulty in lengthening the step (Morris and Iansek, 1996).

Reactive balance performance was associated with several gait adaptability measures: stepping accuracy in the short target condition, number of steps taken to approach the target/obstacle and step length in the walk-through and short/long target conditions. This indicates balance control plays an important role in gait adaptability, particularly for accurate foot placement in a walking task requiring stepping adjustments. Our findings corroborate previous work that has demonstrated the importance of adequate postural adjustments during gait initiation with the goal to clear an obstacle in young adults (Yiou et al., 2016a,b). Previous work has also shown people with PD display inappropriate postural adjustments (reduced anteroposterior center of mass motion and smaller distance between the center of pressure and center of mass) during obstacle crossing (Stegemoller et al., 2012) and prior to step initiation when performing a concomitant attentional task (Tard et al., 2014).

Our finding that reactive balance predicts step length in the walk-through condition suggests participants whose reactive balance is compromised adopt a cautious walking pattern in a cognitively demanding situation that may require step adjustments to negotiate hazards (Caetano et al., 2017). Furthermore, a higher concern about falling was associated with many gait adaptability parameters in the univariate analyses and was an independent predictor of step length at baseline and previous step in the obstacle condition which suggests psychological factors also influence gait performance in situations involving environmental hazards (van Schooten et al., 2019).

Finally, it is surprising that overall freezing of gait was generally not related to the gait adaptability parameters. Participants were tested “on” their usual PD medication thus they are less likely to demonstrate freezing of gait. Also, the task was highly attention demanding with some predictability about when the target or obstacle would appear. People with freezing of gait are known to perform better when external cueing are provided (Ginis et al., 2018), thus the GAT may have worked as a visual cue to the freezers.

### Practical Implications

Our findings demonstrate the importance of considering the context under which walking performance is assessed. A complex interplay of sensorimotor and cognitive abilities are likely required for people with PD to meet everyday challenges associated with walking, such as crossing streets, moving in crowds, etc. Thus, the GAT provides a novel way to explore sensorimotor and cognitive mechanisms involved in complex walking tasks. Our findings also suggest that impaired inhibitory control contributes to stepping errors and may increase fall risk in people with PD. This information may elucidate mechanism as to why people with PD fall and facilitate fall risk assessments and fall prevention strategies for this group. These strategies could involve adding decision-making tasks (e.g., obstacle avoidance) to walking adaptability training. Future studies could also investigate whether gait adaptability measures are associated with prospective falls and whether rehabilitation interventions aimed at improving gait adaptability can prevent falls in people with PD.

### Limitations

We acknowledge certain study limitations. First, our study sample was relatively small, and we conducted multiple comparisons relating to the different outcome measures. It is possible, therefore, that some of the associations uncovered are due to chance. However, similar associations were evident between step length and particular gait adaptability parameters, and are also in line with previous findings in healthy older people (Caetano et al., 2017). Second, the calculation of the Stroop stepping test score used a standard version of the CSRT and not a congruent word/arrow direction as the simpler test condition. It may therefore not represent a “pure” measure of inhibitory control of stepping.

## Conclusion

In conclusion, superior executive function, effective reactive balance and good muscle power were associated with successful gait adaptability in people with PD. Executive function and reactive balance appear particularly important for precise foot placements, and cognitive capacity for step length adjustments for avoiding obstacles.

## Data Availability

The datasets generated for this study are available on request to the corresponding author.

## Ethics Statement

This study was carried out in accordance with the recommendations of The University of Sydney Human Research Ethics Committee with written informed consent from all subjects. All subjects gave written informed consent in accordance with the Declaration of Helsinki. The protocol was approved by the Human Research Ethics Committee at the University of Sydney (Project Number: 2013/207).

## Author Contributions

MC, SL and JM conceived the study objectives and designed the study. MC, NA, JS, SP and CC acquired the data. MC, SL and JM analyzed and interpreted the data. MC drafted the manuscript. All authors were involved with preparation of the manuscript.

## Conflict of Interest Statement

The authors declare that the research was conducted in the absence of any commercial or financial relationships that could be construed as a potential conflict of interest.
